# Supplementary material for: Epithelial Sodium Channel Inhibition by Amiloride Addressed with THz Spectroscopy and Molecular Modeling
Source: Molecules. 2022 May 19;27(10):3271. doi: 10.3390/molecules27103271 (PMC9144217; doi:10.3390/molecules27103271)
Supplement: Supplementary file 1 [file molecules-27-03271-s001.zip › molecules-1687501-supplementary.pdf]

Supplementary material

```
α_hENaC(6WTH)   114 -----------------------------VSLNIN-LNSDKLVFPAVTICTLNPYRYPEIKEELEELDRITEQTLFDLY 162
β_hENaC(6WTH)    78 ----------------------------EVSVSLS-VGFKTMDFPAVTICNASPFKYSKI-KHLLKDLDELMEAVLERI 126
γ_hENaC(6WTH)    80 -----------------------------TVSVSIKVHFRKLDFPAVTICNINPYKYSTVRHLLADLEQETREALKSLY 129
A_cASIC1(4NTX)   45 VVWALCFMGSLALLALVCTNRIQYYFLYPHVTKLDEVAATRLTFPAVTFCNLNEFRF----------SRVTKNDLYH-- 111
B_cASIC1(4NTX)   45 VVWALCFMGSLALLALVCTNRIQYYFLYPHVTKLDEVAATRLTFPAVTFCNLNEFRFSRVTKNDLYHAGELLALLNNRY 123
C_cASIC1(4NTX)   45 VVWALCFMGSLALLALVCTNRIQYYFLYPHVTKLDEVAATRLTFPAVTFCNLNEFRFSRVTKNDLYHAGELLALLNNRY 123
α_xENaC(model)   49 AFWLVLFLVTFGLMYWQFGLLFGQYFSYPVSINLN-VNSDKLPFFAVTVCTLNPYRYKAIQNDLQELDKETQRTLYELY 126
β_xENaC(model)   56 VMWFILTLVFAGLVFWQWGVLILTYLSYGVSVSLS-IGFKTMEFPAVTLCNANPFKYSRV-KPLLKELDELVATALDRI 132
γ_xENaC(model)   55 WIWISLTLCAVAVIFWQCALLLMSYY---SVSASITVTFQKLVYPAVTICNLNPYSYSKVKDRLAALEKETSQTLKNIY 130

α_hENaC(6WTH)   163 KYSST------------------LPHPLQRL-------------------------KIGFQLCNQNKSDCFYQTYSSGV 198
β_hENaC(6WTH)   127 ----------------LAPELNLNFSIWNHTPLVLIDERNPHHPMVLDLFASEKICNAHGC---------------KMA 174
γ_hENaC(6WTH)   130 GFPEPR------------------FSHRIPLLIFDQV------------------------------------VGFQLC 154
A_cASIC1(4NTX)  112 -----------------------AGELLALL-------------------------NNRYEIPDTQTAD------EKQL 136
B_cASIC1(4NTX)  124 ----------------------------------EIPDTQTADEKQLEILQD--------------------------- 141
C_cASIC1(4NTX)  124 EIPDTQ------------------TADEKQLEILQDK------------------------------------ANFR-- 146
α_xENaC(model)  127 KYNSTGVQGWIPNNQRVKRDRAGLPYLLELLPPGSETHRVSRSVIEEELQVKRREWNIGFKLCNETGGDCFYQTYTSGV 205
β_xENaC(model)  133 QFSSQNQGNTFTHNNQTRQNVTLDPALWNHIPLVVIDETDPRNPIIHNIFDNNAVYSKNSSIRNSSEDQTSYSQRYKVA 211
γ_xENaC(model)  131 GFTEPLIRSKRDVGVNVENSTEDIFLKQIPLYRLESVKGSQLVVSDLKTKKRTRMSAKVIHRDAESVQDPGNMVGFKLC 209

α_hENaC(6WTH)   199 DAVREWYRFHYINILSRLPETLPSLE-----EDTLGNFIFACRFNQVSCNQANYSHFHHPMYGNCYTFN--DKNNSNLW 270
β_hENaC(6WTH)   175 MRLCSLNRTQCTFRNFTSATQALTEWYILQATNIFAQVPQQELVEMSYPGEQMILACLFGAEPCNYRNFTSIFYPHYGN 253
γ_hENaC(6WTH)   155 -SNDTSDCATYTFSSGINAIQEWYKLHYMNIMAQVPLEKKINMSYSAEELLVTCFFDGVSCDARNFTLFHHPMHGNCYT 232
A_cASIC1(4NTX)  137 EILQDKANFRNFK-----PKPFNMLEFYDRAGHDIREMLLSCFFRGEQCSPEDFKVVFTR-YGKCYTFNAGQDGKPRL- 208
B_cASIC1(4NTX)  142 ---------KANFRNFKPKPFNMLEFY----------------DRAGHDIREMLLSCFFRGEQCSPEDFKVVF-TRYGK 194
C_cASIC1(4NTX)  147 -----------NFKPKPFNMLEFYD----------------RAGHDIREMLLSCFFRGEQCSPEDFKVVFTR-YGKCYT 197
α_xENaC(model)  206 DAIREWYRFHYINILARVPQE-AAID-----GEQLENFIFACRFNEESCTKANYSSFHHAIYGNCYTFNQNQSDQSNLW 278
β_xENaC(model)  212 MKLCTNNNTQCVYRNFTSGVQALREWYLLQLSSIFSNVPLSGRIDMGFKAEDLILTCLFGGQPCSYRNFTHIYDADYGN 290
γ_xENaC(model)  210 DPKNSSDCTIFTFSSGVNAIQEWYRLHYTNILAKISMEDKIAMGYKADELIVTCFFDGLSCDARNFTLFHHPLYGNCYT 288

α_hENaC(6WTH)   271 MSSMPGINNGLSLMLRAEQNDFIPLLSTV------TGARVMVHGQDEPAFMDDGGFNLRPGVETSISMRKETLDRLGGD 343
β_hENaC(6WTH)   254 CYIFNWGMT-EKALPSANPGTEFGLKLILDIGQEDYVPFLAST------AGVRLMLHEQRSYPFIRDEGIYAMSGTETS 325
γ_hENaC(6WTH)   233 FNNRENET-ILSTSMGGSEYGLQVILYINEEEYNPFLVSS------TGAKVIIHRQDEYPFVEDVGTEIETAMVTSIGM 304
A_cASIC1(4NTX)  209 ITMKGGTGNGLEIMLDIQQDEYLPVWGETDETSFEAGIKVQIHSQDEPPLIDQLGFGVAPGFQTFVSCQEQRLIYLPPP 287
B_cASIC1(4NTX)  195 CYTFNAGQDGKPRLITMKGGTGNGLEIMLDIQQDEYLPVWGETDETSFEAGIKVQIHSQDEPPLIDQLGFGVAPGFQTF 273
C_cASIC1(4NTX)  198 FNAGQDGKPRLITMKGGTGNGLEIMLDIQQDEYLPVWGETDETSFEAGIKVQIHSQDEPPLIDQLGFGVAPGFQTFVSC 276
α_xENaC(model)  279 SSSMPGIKNGLTLVLRTEQHDYIPLLSSV------AGARVLVHGHKEPAFMDDNGFNIPPGMETSIGMKKETINRLGGK 351
β_xENaC(model)  291 CYIFNWGQEGENTMSSANPGADFGLKLVLDIEQGEYLPFLQTT------AAARLILHQQRSFPFVKDLGIYAMPGTETS 363
γ_xENaC(model)  289 FNSAERGN-LLVSSMGGAEYGLKVVLYIDEDEYNPYLSTA------AGAKILVHDQDEYPFIEYLGTELETATETSIGM 360

α_hENaC(6WTH)   344 YGDCTKNGSDVPVENLYPSKYTQQVCIHSCFQESMIKECGCAYIFYPRPQNVEYCDYRKHSSWGYCYYKLQVDFSSDHL 422
β_hENaC(6WTH)   326 IGVLVDKLQRMGEPYSPCTVNGSEVPVQNFYSDYNTTYSIQACLRSCFQDHMIRNCNCGHYLYPLPRGEKYCNNRDFPD 404
γ_hENaC(6WTH)   305 HLTESFKLSEPYSQCTEDGSDVPIRNIYNAAYSLQICLHSCFQTKMVEKCGCAQYSQPLPPAANYCNYQQHPNWMYCYY 383
A_cASIC1(4NTX)  288 WGDCKATTGE------FYDTYSITACRIDCETRYLVENCNCRMVH--MPGDAPYCTPEQYKE----CADPALDFLVEKD 354
B_cASIC1(4NTX)  274 VSCQEQRLIYLPPPWGDCKATT---------GEFYDTYSITACRIDCETRYLVENCNCR--MVHMPGDAPYCTPEQYKE 341
C_cASIC1(4NTX)  277 QEQRLIYLPPPWGDCKATTGE------FYDTYSITACRIDCETRYLVENCNCRMVH--MPGDAPYCTPEQYKE---CAD 344
α_xENaC(model)  352 YSDCSEDGSDVDVKNLFQSEYTEQVCVRSCFQAAMVARCGCGYAFYPLSPGDQYCDYNKHKSWGHCYYKLIIEFTSNKL 430
β_xENaC(model)  364 ISVLVDQLEHMEAPYSSCTVNGSDIPVQNLYAEFNSSYSIQSCLRSCYQEEMVKTCKCAHYQYPLPNGSEYCTNMKHPD 442
γ_xENaC(model)  361 QLTESAKLSDPYSDCTMDGRDVSVENLYNKKYTLQICLNSCFQREMVRSCGCAHYDQPLPNGAKYCNYEEYPSWIYCYF 439

α_hENaC(6WTH)   423 GCFTKCRKPCSVTSYQLSAGYSRWPSVTSQEWVFQMLSRQNNYTVNNKRNGVAKVNIFFKELNYKTNSESPS------- 494
β_hENaC(6WTH)   405 WAHCYSDLQMSVAQRETCIGMCKESCNDTQYKMTISMADWPSEASEDWIFHVLSQERDQ----TLSRKGIVKLNIYFQE 479
γ_hENaC(6WTH)   384 QLHRAFVQEELGCQSVCKEACSFKEWTLTTSLAQWPSVVSEKWLLPVLTWDQGRQVNKKLNKTDLAKLLIFYKDLNQRS 462
A_cASIC1(4NTX)  355 NEYCVCEMPCNVTRYGKELSMVKIPSKASAKY----LAKKYNKSEQYIGENILVLDIFFEALNYETIEQKKAYEVAGLL 429
B_cASIC1(4NTX)  342 ---CADPALDFLVEKDNEYCVCEMPCNVTRYGKELSMVKIPSKASAKYLAK--KYNKSE----QYIGENILVLDIFFEA 411
C_cASIC1(4NTX)  345 PALDFLVEKDNEY-CVCEMPCNVTRYGKELSMVKIPSKASAKYLAK--KYNKSEQ----YIGENILVLDIFFEALNYET 416
α_xENaC(model)  431 GCFTKCRKPCLVSEYQLTAGYSKWPNRVSQDWVLHTLSRQYNLTD---RNGIAKLNIYFEELNYKTILESPTINMAMLL 506
β_xENaC(model)  443 WVPCYYSLRDSVAIRENCISLCQQPCNDTHYKMVISMADWPSAGAEDWIFHVLSYEKDSSHNITVNRNGIVRLNIYFQE 521
γ_xENaC(model)  440 KVYKQFVQEELGCQSACRESCSFKEWTLTRSLAKWPSLNSEEWMLRVLSWELGEKLNKNLTKNDLANLNIFYQDLNSRS 518

α_hENaC(6WTH)       -------------------------------------------
β_hENaC(6WTH)   480 FNYRTIEESAA--------------------------------                                     490
γ_hENaC(6WTH)   463 IMESPA-------------------------------------                                     468
A_cASIC1(4NTX)  430 GDIGGQMGLFIGASILTVLELFDYA------------------                                     454
B_cASIC1(4NTX)  412 LNYETIEQKKAYEVAGLLGDIGGQMGLFIGASILTVLELFDYA                                     454
C_cASIC1(4NTX)  417 IEQKKAYEVAGLLGDIGGQMGLFIGASILTVLELFDYA-----                                     454
α_xENaC(model)  507 SLLGSQWSLWFGSSVLSVVEMLELV------------------                                     531
β_xENaC(model)  522 FNYRSISESEATNVWLLSNLGGQFGFWMGGSVLCIIEFGEII                                      564
γ_xENaC(model)  519 ISESPTYNIVTLLSNFGGQLGLWMSCSMICVLEIIEVFF----                                     557
```

Figure S1. Sequence alignment used in modeling xENaC. The templates were hENaC according to 6WTH structure [13] and cASIC1 according to 4NTX structure [14]. The alignment was generated using Modeller v10.1 [34].

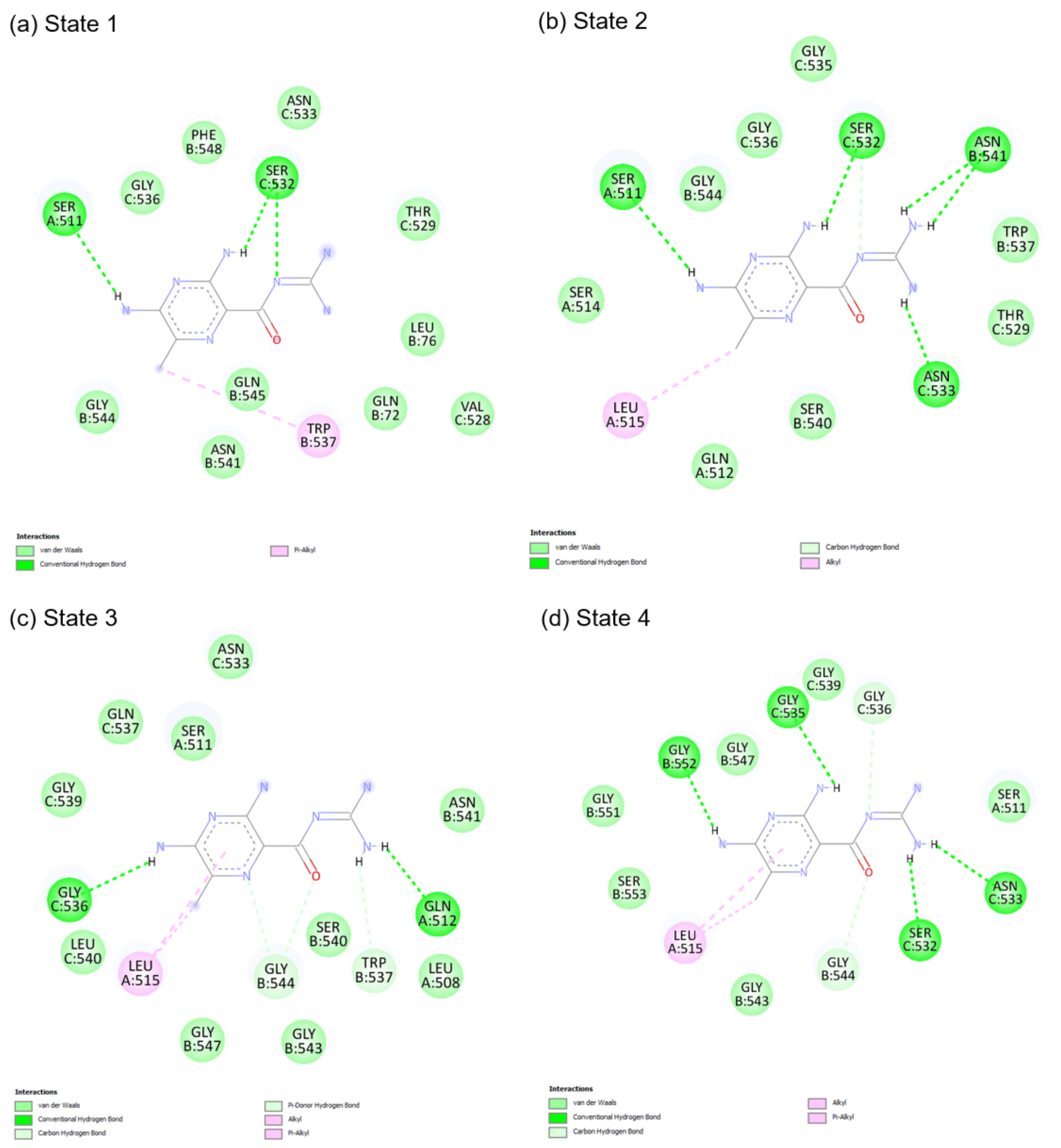

Figure S2. 2D interaction maps of amiloride with the representative structures in the four states: (a) state 1 – after ~1.45 ns of equilibration dynamics (-0.5 ns to the production dynamics), (b) state 2 – after ~2 ns of production dynamics, (c) state 3 – after ~10 ns of production dynamics and (d) state 4 – after ~16 ns of production dynamics. The interactions are represented as follows: dark green for conventional hydrogen bonds (the hydrogen bonds are green dashed lines), light green for van der Waals interactions, very light green for carbon - hydrogen bonds or pi-donor hydrogen bonds, pink for alkyl or pi-alkyl interactions, orange for pi-carbon interactions and red for unfavorable interactions.

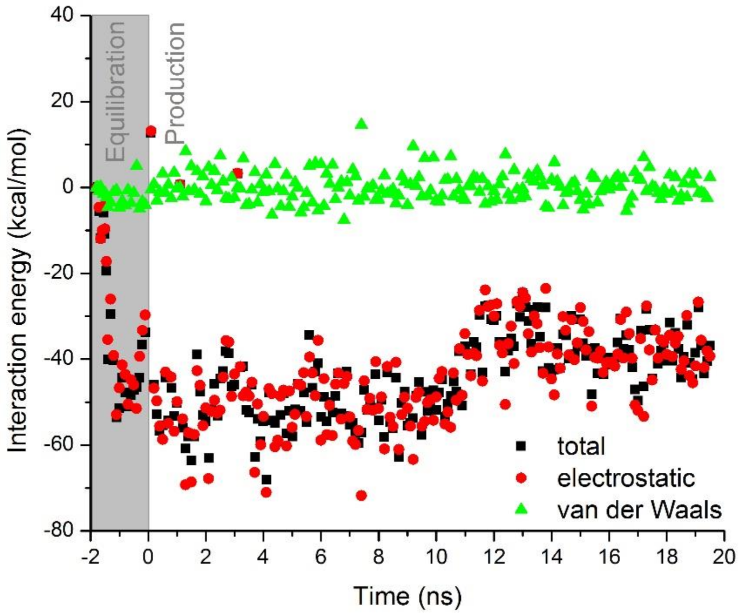

Figure S3. Interaction energies of amiloride with water molecules. Black is used for total interaction energy, red for the electrostatic interaction energy and green for van der Waals interaction energy. The grey background was used to highlight values obtained for the equilibration dynamics and white background is used for values associated with the production dynamics.

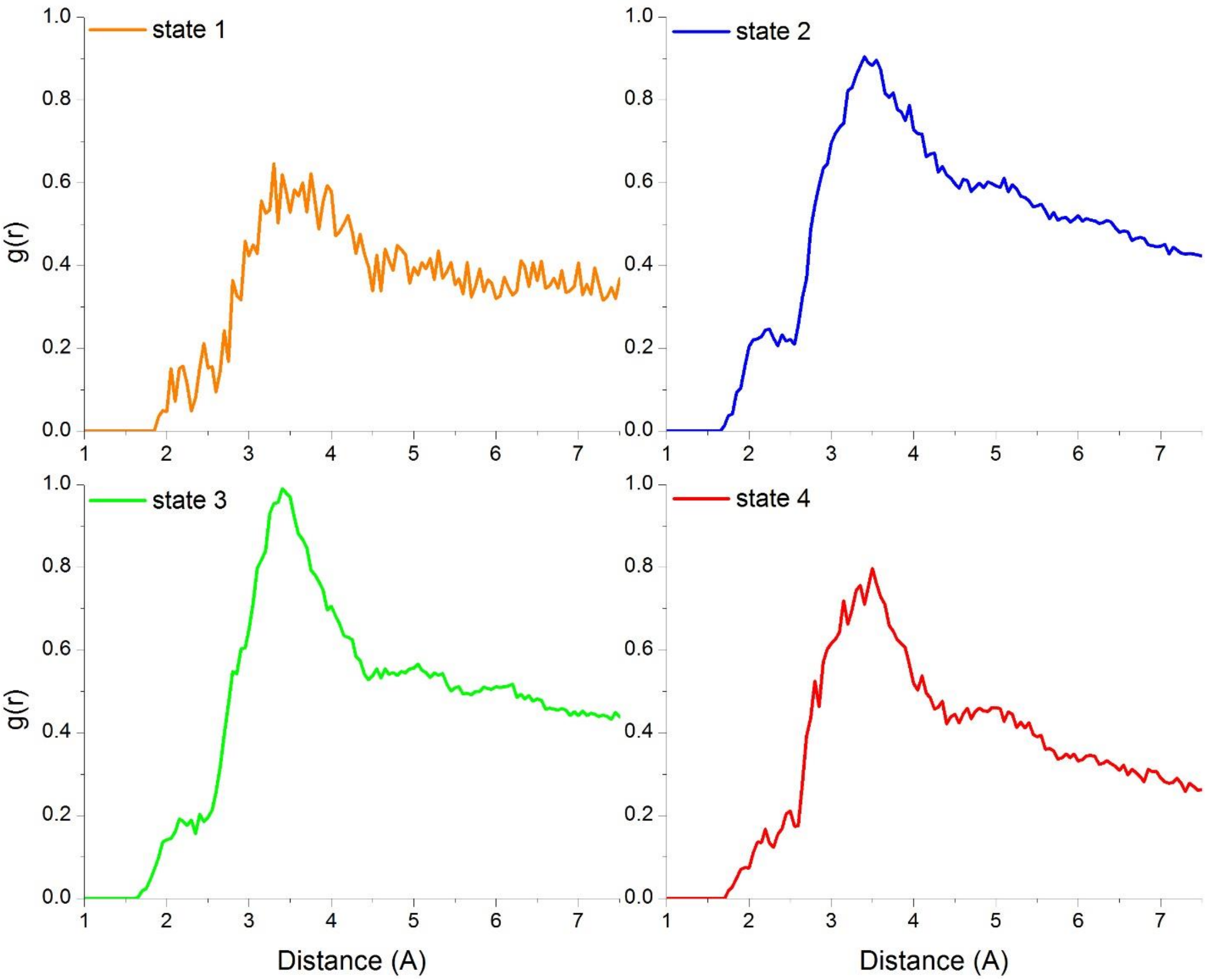

Figure S4. Radial distribution functions (g(r)) of water oxygen atoms around amiloride. The functions were calculated on MD trajectories corresponding to the four states of amiloride, namely state 1 – the last 1.5 ns of equilibration dynamics, state 2 – the range 2 – 6 ns of production MD, state 3 – the range 6-10 ns of production MD, state 4 – the range 12 – 20 ns of production MD. Calculations were performed considering the water molecules on a distance of 7.5 Å around amiloride.

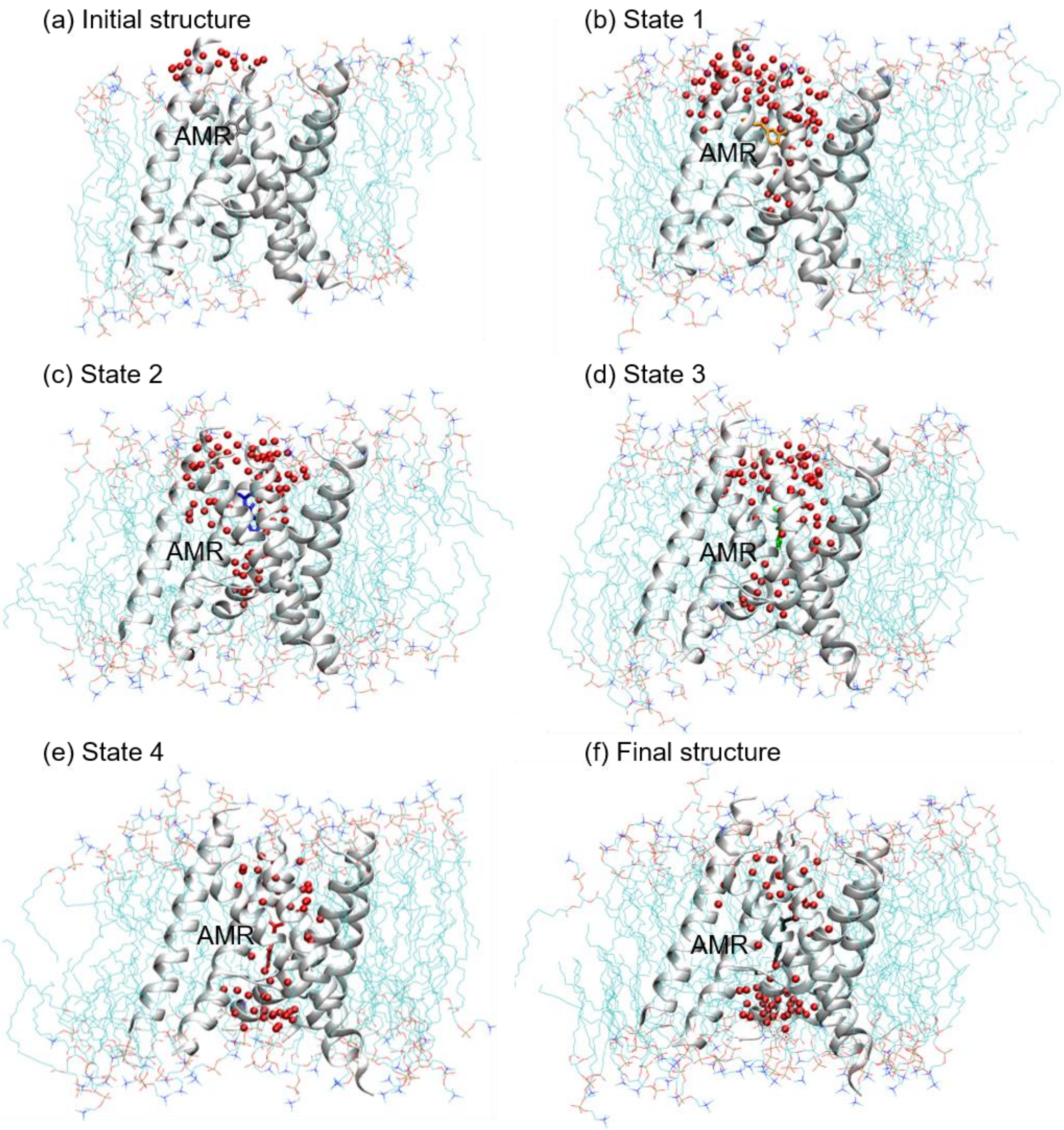

Figure S5. Snapshots of xENaC TM region with bound amiloride. Amiloride is found in the positions associated with the initial structure (a), the four states accessed during the dynamics: after ~1.45 ns of equilibration (b – state 1), after ~2 ns of production dynamics (c – state 2), after ~10 ns of production dynamics (d – state 3), after ~16 ns of production dynamics (e – state 4) and the final structure, after 20 ns of production dynamics (f). The protein is represented in white cartoons. Amiloride is represented as licorice and colored according to its position: grey in (a), orange in (b), blue in (c), green in (d), red in (e) and black in (f). Amiloride coloring scheme is consistent with that in Figure 4 (c). The oxygen atoms from

the water molecules within 10 Å of amiloride are represented as red spheres and lipids within 5 Å of the protein are represented as lines colored according to atom types.
